# Transcription of the Alginate Operon in Pseudomonas aeruginosa Is Regulated by c-di-GMP

**DOI:** 10.1128/spectrum.00675-22

**Published:** 2022-07-11

**Authors:** Ziwei Liang, Morten Rybtke, Kasper Nørskov Kragh, Owen Johnson, Muriel Schicketanz, Yong Everett Zhang, Jens Bo Andersen, Tim Tolker-Nielsen

**Affiliations:** a Costerton Biofilm Center, Department of Immunology and Microbiology, Faculty of Health and Medical Sciences, University of Copenhagen, Copenhagen, Denmark; b Department of Biology, Copenhagen Biocenter, University of Copenhagen, Copenhagen, Denmark; University of Manitoba

**Keywords:** c-di-GMP, alginate, *P. aeruginosa*, transcriptional regulators

## Abstract

Overproduction of the exopolysaccharide alginate contributes to the pathogenicity and antibiotic tolerance of Pseudomonas aeruginosa in chronic infections. The second messenger, c-di-GMP, is a positive regulator of the production of various biofilm matrix components and is known to regulate alginate synthesis at the posttranslational level in P. aeruginosa. We provide evidence that c-di-GMP also regulates transcription of the alginate operon in P. aeruginosa. Previous work has shown that transcription of the alginate operon is regulated by nine different proteins, AmrZ, AlgP, IHFα, IHFβ, CysB, Vfr, AlgR, AlgB, and AlgQ, and we investigated if some of these proteins function as a c-di-GMP effector. We found that deletion of *algP*, *algQ*, *IHFα*, and *IHFβ* had only a marginal effect on the transcription of the alginate operon. Deletion of *vfr* and *cysB* led to decreased transcription of the alginate operon, and the dependence of the c-di-GMP level was less pronounced, indicating that Vfr and CysB could be partially required for c-di-GMP-mediated regulation of alginate operon transcription. Our experiments indicated that the AmrZ, AlgR, and AlgB proteins are absolutely required for transcription of the alginate operon. However, differential radial capillary action of ligand assay (DRaCALA) and site-directed mutagenesis indicated that c-di-GMP does not bind to any of the AmrZ, AlgR, and AlgB proteins.

**IMPORTANCE** The proliferation of alginate-overproducing P. aeruginosa variants in the lungs of cystic fibrosis patients often leads to chronic infection. The alginate functions as a biofilm matrix that protects the bacteria against host immune defenses and antibiotic treatment. Knowledge about the regulation of alginate synthesis is important in order to identify drug targets for the development of medicine against chronic P. aeruginosa infections. We provide evidence that c-di-GMP positively regulates transcription of the alginate operon in P. aeruginosa. Moreover, we revisited the role of the known alginate regulators, AmrZ, AlgP, IHFα, IHFβ, CysB, Vfr, AlgR, AlgB, and AlgQ, and found that their effect on transcription of the alginate operon is highly varied. Deletion of *algP*, *algQ*, *IHFα*, or *IHFβ* only had a marginal effect on transcription of the alginate operon, whereas deletion of *vfr* or *cysB* led to decreased transcription and deletion of *amrZ*, *algR*, or *algB* abrogated transcription.

## INTRODUCTION

Pseudomonas aeruginosa is an opportunistic pathogen which is involved in a variety of infections, including cystic fibrosis (CF) pneumonia, chronic wound infections, catheter-associated urinary tract infections, and ventilator-associated pneumonia (1, 2). In these infections, the bacteria predominantly reside in biofilms and tolerate high doses of antibiotics, and therefore, current treatments of the infections are often not effective (3, 4). Mucoid P. aeruginosa variants overproducing the exopolysaccharide alginate often emerge during chronic CF lung infections (5). Alginate functions as a biofilm matrix component and protects the bacteria against the immune system and antibiotics (3, 6). Knowledge about the molecular mechanisms that are involved in alginate production by P. aeruginosa is highly useful for identification of novel targets for the development of much-needed new medicine against chronic CF lung infections.

Bis-(3′-5′) cyclic dimeric-GMP (c-di-GMP) is a secondary messenger that regulates biofilm formation in a variety of bacteria, including P. aeruginosa (7, 8). A high level of c-di-GMP drives bacteria to form biofilms, whereas reduced c-di-GMP levels promote a planktonic mode of life. In many bacterial species, the level of c-di-GMP is controlled by several different diguanylate cyclase (DGC) enzymes that catalyze formation of c-di-GMP, and several different phosphodiesterase (PDE) enzymes that catalyze degradation of c-di-GMP (7). The DGC and PDE enzymes often have sensory domains and are thought to adjust the c-di-GMP level in response to environmental cues. c-di-GMP is known to regulate the production of biofilm matrix components at the transcriptional, translational, and posttranslational levels (7, 8).

Besides alginate, P. aeruginosa can produce the exopolysaccharides Pel and Psl, which also serve as biofilm matrix components (9–13). The synthesis in P. aeruginosa of these exopolysaccharides is regulated by c-di-GMP at various levels (14). c-di-GMP has been shown to regulate synthesis of alginate at the posttranslational level in P. aeruginosa (15). The Alg44 protein is part of the alginate synthase complex, and upon binding of c-di-GMP to its PilZ domain, alginate polymerization is activated (15). Psl synthesis is regulated by c-di-GMP at the transcriptional level (16), whereas Pel synthesis is regulated by c-di-GMP at both the transcriptional and posttranslational levels (15, 17). At low c-di-GMP levels, the transcriptional regulator FleQ represses transcription of the *pel* operon, whereas at high c-di-GMP levels, FleQ binds c-di-GMP and activates transcription of the *pel* operon (18). At the posttranslational level, c-di-GMP binds to the synthase protein PelD and activates polymerization of Pel (19, 20).

In this study, we asked whether the multitiered c-di-GMP regulation observed for Pel synthesis also applies to regulation of alginate synthesis in P. aeruginosa. We provide evidence that in addition to posttranslational regulation, transcription of the alginate operon is also regulated by c-di-GMP, and we attempted to uncover the underlying mechanistic basis.

## RESULTS AND DISCUSSION

### Construction and characterization of a fluorescent monitor that gauges transcription of the alginate operon.

In order to determine the level of transcription of the alginate operon, we fused the promoter of *algD*, the first gene of the alginate operon, to *gfp*. The *algD* promoter contains binding sites for a variety of transcriptional regulators, and the *algD* gene contains a large 5′ untranslated region (5′ UTR), suggesting significant posttranscriptional regulation (21). We fused the entire *algD* promoter, including the 5′ UTR, to *gfp*, essentially creating a transcriptional *algD-gfp* fusion. The *algD-gfp* construct was cloned into a mini-Tn*7* vector (pTn*7*::*algD-gfp*) suited for chromosomal insertion at a neutral genetic locus. The single-copy monitor was chosen to mitigate any confounding titration effects on transcriptional regulation of the promoter that could arise from employing a multicopy plasmid-based monitor.

**FIG 1:**
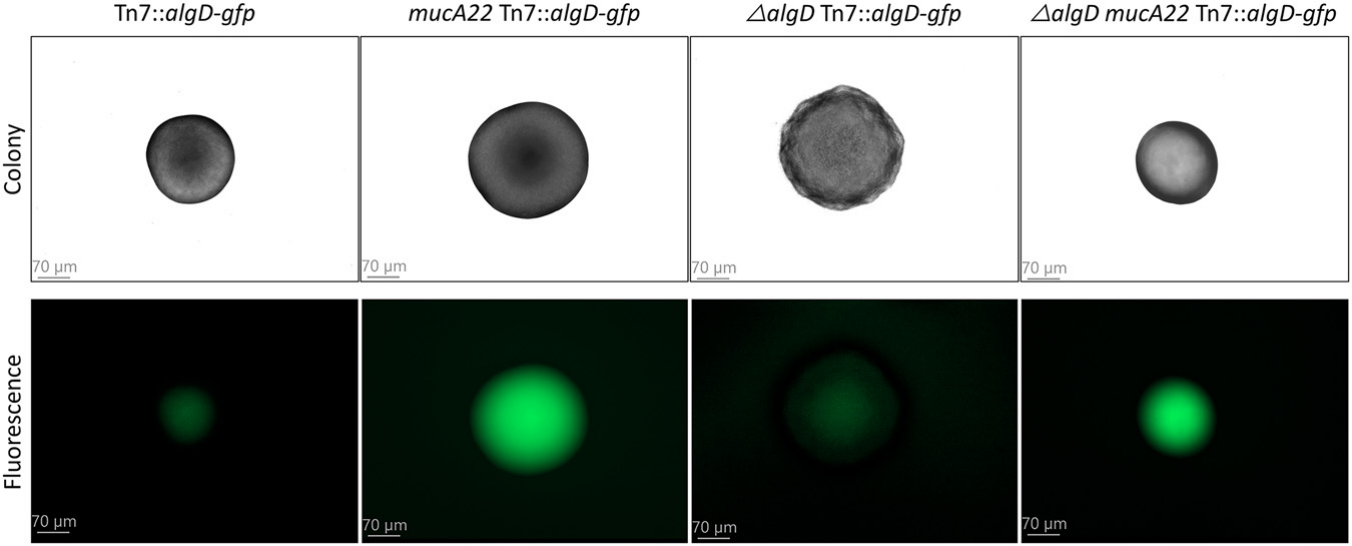
Characterization of the fluorescent monitor that gauges transcription of the alginate operon. The upper row shows colonies formed by the Tn*7*::*algD*-*gfp*, *mucA22* Tn*7*::*algD*-*gfp*, Δ*algD* Tn*7*::*algD*-*gfp*, and Δ*algD mucA22* Tn*7*::*algD*-*gfp* P. aeruginosa PAO1 derivatives, whereas the lower row shows the fluorescence emitted by the colonies. Size bars are 70 μm.

The *algD-gfp* fusion was inserted in the nonmucoid PAO1 wild-type strain and an isogenic mucoid strain harboring the clinically relevant *mucA22* allele. Transcription of the alginate operon is repressed in the wild-type PAO1 strain due to sequestering of the AlgU sigma factor by the anti-sigma factor MucA, whereas the *mucA22* mutant encodes a C-terminally truncated version of MucA that does not sequester AlgU (21, 22). Functionality of the alginate transcription reporter was verified by fluorescence microscopy of the two strains, with the *mucA22* Tn*7*::*algD-gfp* monitor strain displaying bright fluorescence in contrast to the dim fluorescence observed for the wild-type monitor strain (Fig. 1). Since many of the results in this study are based on fluorescence measurements in planktonic cultures, we deleted *algD* in the monitor strains to eliminate alginate production and avoid extensive clumping during cultivation. The resulting Δ*algD* Tn*7*::*algD-gfp* and Δ*algD mucA22* Tn*7*::*algD-gfp* strains displayed colony fluorescence similar to that of the alginate-proficient monitor strains (Fig. 1).

### c-di-GMP positively regulates transcription of the alginate operon.

P. aeruginosa *mucA* mutants are prevalent in chronic CF infections (5), and therefore, we found it of interest to study factors beyond MucA that are involved in transcriptional regulation of the alginate operon. To enable manipulation of the cellular level of c-di-GMP, we inserted the DGC-encoding *yfiN* gene (also termed *tpbB* or PA1120) and the PDE-encoding PA2133 gene under the control of the arabinose-inducible P*_BAD_* promoter in the φCTX attachment site of the Δ*algD mucA22* Tn*7*::*algD-gfp* strain. We found, however, that the arabinose-induced increase of the c-di-GMP level in the Δ*algD mucA22* Tn*7*::*algD-gfp* CTX::*araC*-P*_BAD_*-PA1120 strain caused an undesired hyperaggregation due to overproduction of biofilm matrix components (data not shown). To reduce aggregation, we deleted the *pelA* and *pslBCD* genes in the Δ*algD mucA22* Tn*7*::*algD-gfp* strain, rendering it deficient for production of the Pel and Psl polysaccharides. The inducible DGC and PDE constructs were subsequently inserted to create the Δ*pel* Δ*psl* Δ*algD mucA22* Tn*7*::*algD-gfp* CTX::*araC*-P*_BAD_*-PA1120 DGC strain and the Δ*pel* Δ*psl* Δ*algD mucA22* Tn*7*::*algD-gfp* CTX::*araC*-P*_BAD_*-PA2133 PDE strain.

**FIG 2:**
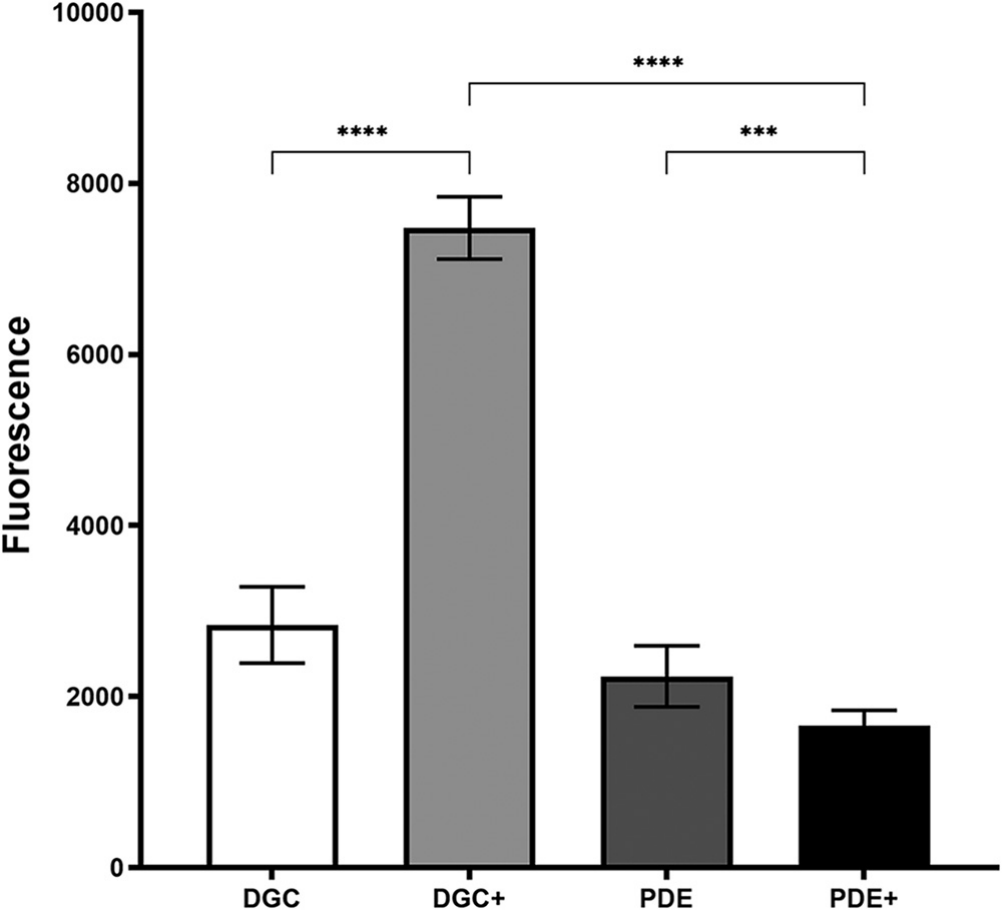
c-di-GMP positively regulates transcription of the alginate operon. Shown is fluorescence of late-log-phase cultures of the Δ*pel* Δ*psl* Δ*algD mucA22* Tn*7*::*algD*-*gfp* CTX::*araC*-P*_BAD_*-PA1120 strain without arabinose (DGC), the Δ*pel* Δ*psl* Δ*algD mucA22* Tn*7*::*algD*-*gfp* CTX::*araC*-P*_BAD_*-PA1120 strain with arabinose (DGC+), the Δ*pel* Δ*psl* Δ*algD mucA22* Tn*7*::*algD*-*gfp* CTX::*araC*-P*_BAD_*-PA2133 strain without arabinose (PDE), and the Δ*pel* Δ*psl* Δ*algD mucA22* Tn*7*::*algD*-*gfp* CTX::*araC*-P*_BAD_*-PA2133 strain with arabinose (PDE+). Means and standard deviations (bars) of 15 replicates are shown. Significance levels are indicated as follows: ***, *P* < 0.001, and ****, *P* < 0.0001.

Subsequently, a microtiter assay was carried out to determine the effect of the cellular c-di-GMP level on the transcription of the alginate operon as indicated by the fluorescence level of the monitor strains. The experiments were carried out both with and without arabinose induction of the P*_BAD_* promoters. As shown in Fig. 2, the fluorescence of the arabinose-induced high-c-di-GMP-level strain (DGC+) was significantly higher than that of the uninduced DGC strain as well as the induced and uninduced PDE strains. Moreover, the fluorescence of the arabinose-induced PDE strain was significantly lower than that of the uninduced PDE strain. These data suggest that c-di-GMP positively regulates transcription of the alginate operon.

The deletion of the *psl* and *pel* genes in our bioreporter strains could in principle affect c-di-GMP signaling, since production of the Psl and Pel polysaccharides is connected to c-di-GMP pathways (16–18, 23). However, we believe that use of the inducible *araC*-P*_BAD_*-PA1120 and *araC*-P*_BAD_*-PA2133 constructs overrides such effects. Yet to corroborate our results, we constructed the strain *mucA22* CTX::*araC*-P*_BAD_*-PA1120 and used quantitative real-time PCR (qRT-PCR) to assess the effects of high and low c-di-GMP content on transcription of the alginate operon. As shown in Fig. S1 in the supplemental material, our qRT-PCR analysis showed that transcription of the alginate operon is positively regulated by c-di-GMP also in the strain with intact *psl* and *pel* genes. The results also exclude that our findings could be caused solely by a hypothetical c-di-GMP-mediated regulation at the 5′ UTR of the *algD* promoter.

We noticed that cultures of the arabinose-induced DGC strain displayed a lower optical density than the uninduced DGC strain at the time point where the fluorescence measurements were conducted (data not shown). However, determination of CFU from the cultures at this specific time point implied no significant difference in growth between the arabinose-induced and uninduced DGC cultures (Fig. S2). Microscopy of culture samples revealed that the arabinose-induced DGC strain was growing as small aggregates, whereas the uninduced DGC strain mainly grew as single cells (Fig. S2), which might explain the difference in optical density. Because of these findings, we chose not to normalize our fluorescence measurements against the optical density of the cultures. Interestingly, we have previously observed that if the c-di-GMP level is increased by means of a *wspF* mutation, which results in activation of the WspR DGC (16), cultures of a P. aeruginosa Δ*pel* Δ*psl* strain do not display clumping (24). It is possible that high-level expression of YfiN, used in the present study to increase the cellular c-di-GMP level, results in highly increased production of c-di-GMP-regulated factors, such as CdrA adhesin or Cup fimbriae, that can cause aggregation independent of the matrix exopolysaccharides.

### The role of known regulators in c-di-GMP-mediated transcriptional regulation of the alginate operon.

**FIG 3:**
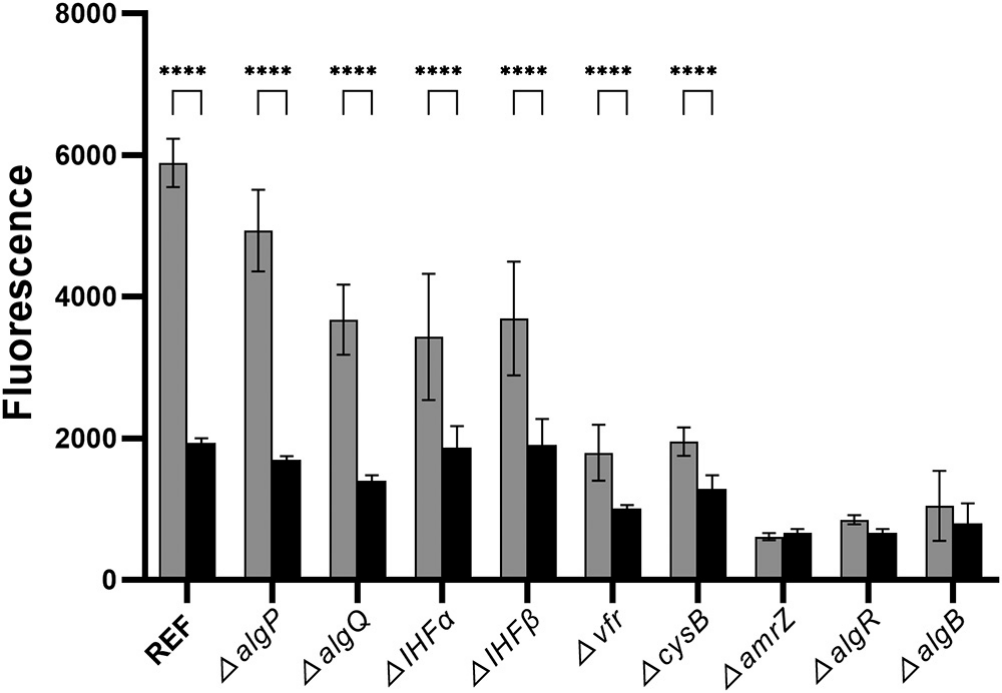
The role of known regulators in c-di-GMP-mediated transcriptional regulation of the alginate operon. The two bars labeled REF show fluorescence of late-log-phase cultures of the Δ*pel* Δ*psl* Δ*algD mucA22* Tn*7*::*algD*-*gfp* CTX::*araC*-P*_BAD_*-PA1120 strain with arabinose (gray bars) and the Δ*pel* Δ*psl* Δ*algD mucA22* Tn*7*::*algD*-*gfp* CTX::*araC*-P*_BAD_*-PA2133 strain with arabinose (black bars). The following bars show fluorescence of late-log cultures of the same two background strains with additional gene deletions as indicated. Means and standard deviations (bars) of 15 replicates are shown. Significance levels are indicated as follows: ****, *P* < 0.0001.

Subsequently, we investigated the role of known regulators in c-di-GMP-mediated transcriptional regulation of the alginate operon. Previous work has shown that transcription of the alginate operon is regulated by nine different proteins, AmrZ (25, 26), AlgP (27), IHFα (28), IHFβ (28), CysB (29), Vfr (21), AlgR (30), AlgB (31), and AlgQ (32), of which all but Vfr and AlgQ have been shown to bind to the *algD* promoter. We hypothesized that c-di-GMP-mediated regulation of alginate transcription might occur through one of these nine regulators. To test this hypothesis, we deleted each of these nine regulators from our DGC and PDE strains to investigate if they are involved in c-di-GMP-mediated regulation. The rationale was that if transcription of the alginate operon is only partially dependent on a transcription factor, and if deletion of the transcription factor eliminates the effect of the c-di-GMP level on transcription, then that particular transcription factor functions as a c-di-GMP effector. As shown in Fig. 3, the deletion of *algP*, *algQ*, *IHFα*, and *IHFβ* had only a marginal effect on the transcription of the alginate operon, and the dependency of the level of c-di-GMP was maintained. Our results with the *algP* mutant are in accordance with a recent study showing that deletion of *algP* in PAO1 (nonmucoid) and PDO300 (mucoid) did not result in reduction of the alginate level (33). On the contrary, deletion of *vfr* and *cysB* led to decreased transcription of the alginate operon, and the dependence of the c-di-GMP level was less pronounced (Fig. 3), indicating that Vfr and CysB are partially required for c-di-GMP-mediated regulation of alginate operon transcription. Deletion of the *amrZ*, *algR*, and *algB* genes led to a highly decreased transcription of the alginate operon, which apparently occurred independent of the c-di-GMP level (Fig. 3). However, if the three proteins are absolutely required for transcription of the alginate operon, the experiments could not reveal a role for c-di-GMP in transcription. To exclude the effect of background fluorescence, we constructed deletion mutants of *amrZ*, *algR*, and *algB* in the parent DGC and PDE strains without the transcriptional *algD-gfp* reporters. Subtraction of background fluorescence indicated that the AmrZ, AlgR, and AlgB proteins are absolutely required for transcription of the alginate operon (Fig. S3), and therefore, our experiments could not reveal if the proteins have a role in c-di-GMP-mediated transcriptional regulation of the alginate operon.

### The AmrZ, AlgR, and AlgB proteins do not bind c-di-GMP *in vitro*.

**FIG 4:**
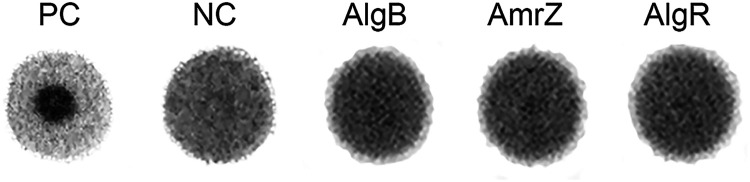
The AmrZ, AlgR, and AlgB proteins do not bind c-di-GMP *in vitro*. DRaCALA spots of binding of ^32^P-c-di-GMP to the negative control (NC), positive control (PC), and the purified AlgB, AmrZ, and AlgR proteins are shown. The NC and PC are whole-cell lysates of E. coli MG1655 cells expressing the empty vector pCA24N or pCA24N-ilvH encoding the c-di-GMP-binding IlvH protein, respectively.

Our work described above indicated that transcription of the alginate operon is absolutely dependent on the transcriptional regulators AmrZ, AlgR, and AlgB, and the experiments could not reveal if any of the proteins have a role in the dependency of transcription on the cellular c-di-GMP level. We employed a differential radial capillary action of ligand assay (DRaCALA) to investigate if c-di-GMP binds to any of the AmrZ, AlgR, and AlgB proteins. The *amrZ*, *algR*, and *algB* genes were cloned into Escherichia coli and the AmrZ, AlgR, and AlgB proteins were purified. Subsequently, the ability of immobilized protein to reduce migration of radioactively labeled c-di-GMP was assayed. If c-di-GMP binds to the tested protein, instead of diffusing, c-di-GMP will be retained at the protein, resulting in a black dot in the center of the spot (34). Whole-cell lysates containing the E. coli IlvH protein overexpressed from the plasmid pCA24N-ilvH were used as the positive control, since the IlvH protein is known to bind c-di-GMP (35), and a whole-cell lysate with an empty vector, pCA24N (36), was used as the negative control. Unlike the positive control, the tested proteins showed no significant black dot in the spot center but showed instead a uniform spot that resulted from freely diffusing c-di-GMP (Fig. 4). This indicates that none of the three proteins bound c-di-GMP *in vitro* and that none of these proteins function as c-di-GMP effectors.

### Site-directed mutagenesis does not reveal a role of AlgB in c-di-GMP-mediated transcriptional regulation of the alginate operon.

**FIG 5:**
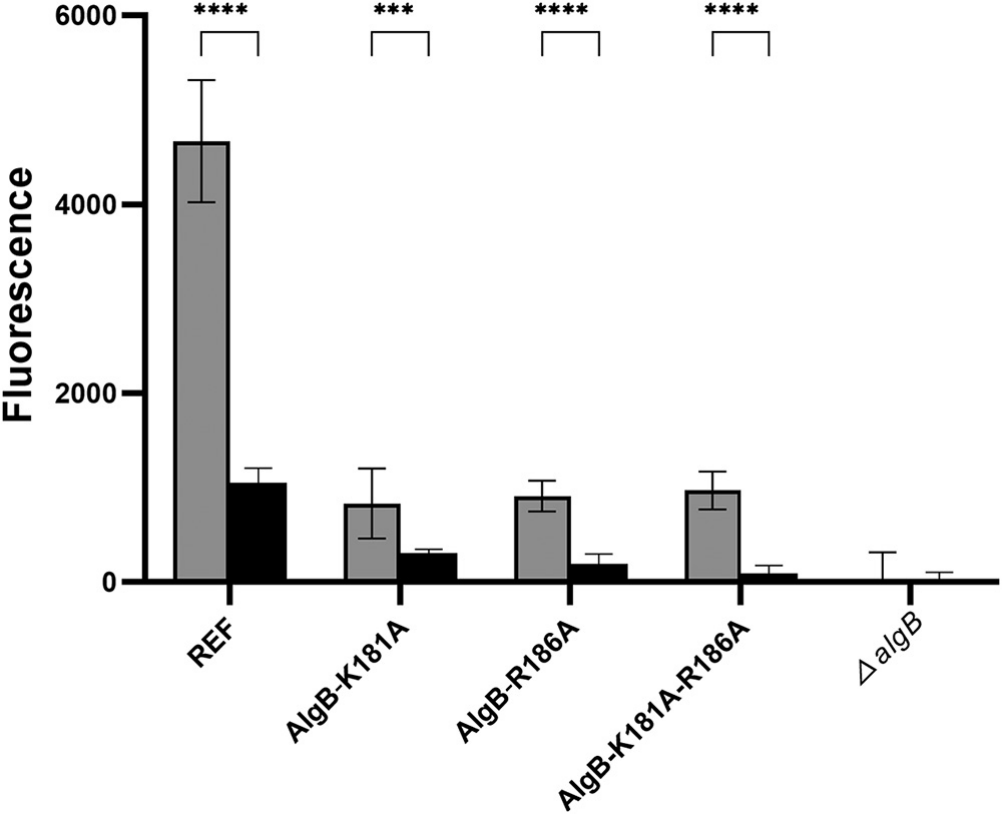
Site-directed mutagenesis does not reveal a role for AlgB in c-di-GMP-mediated transcriptional regulation of the alginate operon. The two bars labeled REF show fluorescence of late-log cultures of the Δ*pel* Δ*psl* Δ*algD mucA22* Tn*7*::*algD*-*gfp* CTX::*araC*-P*_BAD_*-PA1120 strain with arabinose (gray bar) and the Δ*pel* Δ*psl* Δ*algD mucA22* Tn*7*::*algD*-*gfp* CTX::*araC*-P*_BAD_*-PA2133 strain with arabinose (black bar). The following bars show fluorescence of late-log-phase cultures of the same two background strains with additional point mutations or gene deletions as indicated. Fluorescence of cultures of the corresponding strains without the Tn*7*::*algD*-*gfp* fusion are withdrawn. Means and standard deviations (bars) of 9 replicates are shown. Significance levels are indicated as follows: ****, *P* < 0.0001.

The FleQ protein is an established c-di-GMP effector in P. aeruginosa (17). Sequence analysis indicates that AlgB and FleQ belong to the same Ntrc family of enhancer-binding transcriptional regulators. Pair-wise alignment of the amino acid sequences showed that the two proteins have a high degree of sequence similarity (data not shown). Furthermore, FleQ is known to bind c-di-GMP at residues K180 and R185 (37, 38), which correspond to residues K181 and R186, respectively, of AlgB. Because of the similarity of AlgB and FleQ, we decided to use site-directed mutagenesis to investigate if AlgB binds c-di-GMP *in vivo*, although our DRaCALA experiments indicated that AlgB does not bind c-di-GMP *in vitro*. To this end, we constructed K181A and R185A single and double amino acid substitutions in AlgB. Compared to the DGC and PDE strains encoding wild-type AlgB, the strains encoding the single- and double-point mutants of AlgB generally displayed lower transcription levels of the alginate operon (Fig. 5). However, the transcriptional levels were still significantly different between the high- and low-c-di-GMP-level strains. This result indicates that the AlgB protein does not bind c-di-GMP *in vivo*.

### Deletion of the *algR* gene drastically reduces readout from the pCdrA-gfp bioreporter.

**FIG 6:**
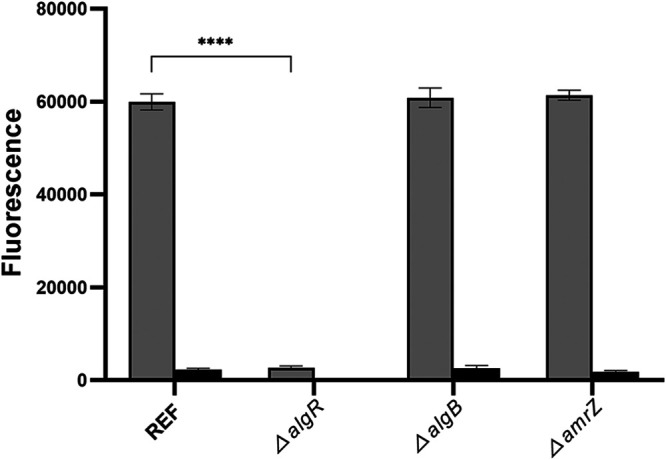
Deletion of the *algR* gene drastically reduces readout from the pCdrA-gfp bioreporter. The two bars labeled REF show fluorescence of late-log-phase cultures of the Δ*pel* Δ*psl* Δ*algD mucA22* CTX::*araC*-P*_BAD_*-PA1120/pCdrA-gfp strain with arabinose (gray bar) and the Δ*pel* Δ*psl* Δ*algD mucA22* CTX::*araC*-P*_BAD_*-PA2133/pCdrA-gfp strain with arabinose (black bar). The following bars show fluorescence of late-log cultures of the same two background strains with additional gene deletions as indicated. Fluorescence of cultures of the corresponding strains without the pCdrA-gfp plasmid are withdrawn. Means and standard deviations (bars) of 9 replicates are shown. Significance levels are indicated as follows: ****, *P* < 0.0001.

We noted that unlike all the other DGC strains, our Δ*algR* Δ*pel* Δ*psl* Δ*algD mucA22* Tn*7*::*algD-gfp* CTX::*araC*-P*_BAD_*-PA1120 strain did not display reduced optical density in cultures supplemented with arabinose (data not shown). This could indicate that the *algR* mutation somehow prevents that induction of the P*_BAD_*-PA1120 fusion results in a high level of c-di-GMP. To investigate this hypothesis, we sought to assess the c-di-GMP level in a selection of our mutant strains. To this end, we employed a fluorescent c-di-GMP reporter, which is based on a fusion between the c-di-GMP-regulated *cdrA* promoter and *gfp* (39). We deleted the *algR*, *algB*, and *amrZ* genes in the Δ*pel* Δ*psl* Δ*algD mucA22* CTX::*araC*-P*_BAD_*-PA1120 and Δ*pel* Δ*psl* Δ*algD mucA22* CTX::*araC*-P*_BAD_*-PA2133 background strains, which do not contain the Tn*7*::*algD-gfp* alginate transcription reporter. Subsequently, we transformed the background and mutant strains with the plasmid-based pCdrA-gfp c-di-GMP reporter. We then determined the readout from the pCdrA-gfp bioreporter in these strains with arabinose induction of either the P*_BAD_*-PA1120 or P*_BAD_*-PA2133 fusion. As shown in Fig. 6, the *algR* mutation prevents that induction of the P*_BAD_*-PA1120 fusion results in a high readout from the pCdrA-gfp bioreporter. A low level of c-di-GMP in the Δ*algR* DGC strain could contribute to the low level of alginate operon transcription observed in this strain. An alternative explanation is that AlgR is necessary for transcription of the *cdrA-gfp* fusion. In that case, clumping of the arabinose-induced DGC strains could be caused by the adhesin CdrA, which would not be expressed in the Δ*algR* DGC strain. Evidence that AlgR promotes synthesis of c-di-GMP by inducing transcription of the *mucR* gene encoding a DGC has previously been presented (40). In that study, both a *cdrA-lux* fusion and mass spectrometry measurements were used to show that an *algR* mutant has reduced content of c-di-GMP compared to that of the wild type. Notably, the c-di-GMP level indicated by the *cdrA-lux* fusion strain correlated with the c-di-GMP level obtained by mass spectrometry measurements, arguing against a role for AlgR in regulation of the *cdrA* promoter. However, our findings with the *algR* mutant are subject to further investigation in our laboratory.

In two previous studies, it was found that an *amrZ* mutation caused elevated levels of c-di-GMP by derepressing the DGC-encoding genes PA4843 and *gcbA* (41, 42). In our study, however, assessment of *cdrA-gfp*-mediated fluorescence did not indicate an increase of the c-di-GMP content in our *amrZ* mutant.

### Conclusions.

In the present study, we have constructed and characterized a fluorescent monitor that gauges transcription of the alginate operon in P. aeruginosa. We engineered the alginate monitor strain with P*_BAD_*-PA1120 and P*_BAD_*-PA2133 fusions so that we could increase or decrease the cellular c-di-GMP content via arabinose induction. By employing these engineered strains, we demonstrated that c-di-GMP positively regulates transcription of the alginate operon in P. aeruginosa. Previous work has shown that transcription of the alginate operon is regulated by nine different proteins, AmrZ, AlgP, IHFα, IHFβ, CysB, Vfr, AlgR, AlgB, and AlgQ, and we attempted to reveal if some of these proteins function as a c-di-GMP effector. We found that deletion of *algP*, *algQ*, *IHFα*, and *IHFβ* had only a marginal effect on the transcription of the alginate operon. Deletion of *vfr* and *cysB* led to decreased transcription of the alginate operon, and the dependence of the c-di-GMP level was reduced, indicating that Vfr and CysB could be partially required for c-di-GMP-mediated regulation of alginate operon transcription. However, this possibility should be investigated further before firm conclusions are drawn. Our experiments indicated that the AmrZ, AlgR, and AlgB proteins are absolutely required for transcription of the alginate operon, and consequently, the experiments could not reveal if the proteins have a role in c-di-GMP-mediated transcriptional regulation of the alginate operon. However, DRaCALA assays indicated that c-di-GMP does not bind to any of the AmrZ, AlgR, and AlgB proteins *in vitro*, and site-directed mutagenesis indicated that c-di-GMP does not bind to AlgB *in vivo*. Finally, our experiments indicate that induction of the P*_BAD_*-PA1120 fusion does not result in a high readout from the pCdrA-gfp bioreporter in an *algR* mutant, a finding that is subject to further study in our laboratory.

## MATERIALS AND METHODS

### Bacterial strains, plasmids, primers, and growth media.

The P. aeruginosa and Escherichia coli strains used in this study are listed in Table S1. The growth media employed for propagation of the strains were either ABTrace medium (43), lysogeny broth (LB; 10 g/L of tryptone [grade LP0042T; Oxoid, United Kingdom], 5 g/L of yeast extract [grade LP0021T; Oxoid], and 10 g/L of NaCl), no-NaCl lysogeny broth (10 g/L of tryptone [grade LP0042T; Oxoid] and 5 g/L of yeast extract [grade LP0021T; Oxoid]), or MTB medium (43) supplemented with 33.7 mM Na_2_HPO_4_, 22 mM KH_2_PO_4_, 1 μM FeCl_3_, 10 mM KNO_3_, 150 μM Na_2_SO_4_, and 55.6 mM glucose. The rationale behind employment of no-NaCl LB medium is that SacB-based counterselection by means of sucrose selection is more efficient in the absence of NaCl. When necessary, growth media were supplemented with the following antibiotics: gentamicin, 15 μg/mL for E. coli and 60 μg/mL for P. aeruginosa; ampicillin, 100 μg/mL for E. coli; carbenicillin, 200 μg/mL for P. aeruginosa; kanamycin, 35 μg/mL for E. coli; and chloramphenicol, 6 μg/mL for E. coli. Plasmids and primers used in this study are listed in Tables S2 and S3, respectively.

### Standard molecular methods.

Genomic DNA (gDNA) was purified using the DNeasy blood and tissue kit (Qiagen, Denmark), plasmids were purified using the QIAprep Spin miniprep kit (Qiagen), and the Wizard SV gel and PCR cleanup system (Promega) was applied for purification of PCR products and DNA fragments excised from agarose gels. PCR amplification was conducted using Phusion polymerase (Thermo Scientific, Denmark) as recommended by the manufacturer.

### Reporter constructs.

Construction of pTn*7*::*algD-gfp* was carried out as follows. Initially, the *algD* promoter (P-*algD*), including the 5′ UTR, was amplified using primers P-algD_F and P-algD_R containing a KpnI restriction site and *gfp* complementary 3′ overhangs, respectively. *gfp* was amplified from pCdrA-gfp using primers gfp_F (P-algD) and gfp_R (P-algD), with the resulting fragment harboring a HindIII restriction site immediately downstream of the coding sequence. The P-*algD* transcriptional fusion was subsequently amplified using splicing by overhang extension PCR (SOE PCR) with primers P-algD_F and gfp_R (P-algD) using the P-*algD* and *gfp* fragments as templates. The fusion was then digested using KpnI and HindIII and ligated into similarly digested pUC18-miniTn*7*T-Gm to give pTn*7*::*algD*-*gfp*. Finally, the structure of pTn*7*::*algD-gfp* was verified by sequencing using the following primers as sequencing primers: Tn*7*L-in, Seq-F-algD, and Gfp-seq-int(+).

### Construction of *algD*-*gfp* reporter strains.

PAO1 strains carrying the *algD*-*gfp* fusion of pTn*7*::*algD-gfp* in their chromosomal Tn*7* insertion sites were constructed by four-parental mating between E. coli DH5α/pTn*7*::*algD*-*gfp* (donor), E. coli HB101/pRK600 (helper), E. coli SM10-λpir/pUX-BF13 (helper) (provider of the Tn*7* transposase) (44), and the appropriate P. aeruginosa strain (recipient) as described previously by Koch and coworkers (45). Briefly, an 18-h-old culture of the recipient P. aeruginosa strain (propagated in LB at 37°C) was diluted 2-fold into 42°C prewarmed LB medium and kept at 42°C for 4 h. Then a mating solution containing a 1:1:1:1 mixture of the heat-treated recipient P. aeruginosa culture and late-exponential-phase cultures of the donor and helper strains was prepared and subsequently spotted onto an LB plate. Following 20 h of mating at 30°C, the resulting mating spot was resuspended in 1 mL of 0.9% NaCl, and transconjugants were selected on ABTrace plates (ABTrace medium [43] solidified with 1.5% (15 g/L) agar (Agar Bacteriological, grade LP0011T; Oxoid, United Kingdom) supplemented with 10 mM sodium citrate, 10 mM FeCl_3_, and 60 μg/mL of gentamicin). Next, the gentamicin marker flanked by FRT sites in the Tn*7*::*algD*-*gfp* cassette located in the resulting transconjugant was excised by employment of the pFlp2 plasmid and subsequent sucrose selection as outlined by Hoang et al. (46). Finally, the chromosomal location of the *algD*-*gfp* fusion in the resulting monitor strain was verified by PCR using the primer pair Tn7R109 and Tn7glmS3.

### Construction of DGC or PDE inducible strains.

To obtain P. aeruginosa strains exhibiting arabinose-inducible expression of the DGC PA1120, the *araC*-P*_BAD_*-PA1120 expression cassette of pENTRminiCTX2-P*_BAD_*-PA1120 (39) was inserted into the chromosome of the P. aeruginosa strain of choice using the three-step protocol described by Andersen and coworkers (47). In step 1, a transconjugant with plasmid pENTRminiCTX2-P*_BAD_*-PA1120 inserted into the chromosomal φ CTX *attB* site was constructed by three-parental mating using E. coli DH5α/pENTRminiCTX2-P*_BAD_*-PA1120 as the donor, E. coli HB101/pRK600 as the helper, and the P. aeruginosa strain of choice as the recipient. In step 2, the created transconjugant was transformed with plasmid pFLP2 (encoding Flp recombinase) (46) to obtain transformants in which the FRT-flanked plasmid backbone of pENTRminiCTX2-P*_BAD_*-PA1120 has been excised by the Flp recombinase. In step 3, the resulting P. aeruginosa strain containing one chromosomal copy of the *araC*-P*_BAD_*-PA1120 expression cassette was cured for plasmid pFLP2 using sucrose-based counterselection.

Finally, the chromosomal location of the *araC*-P*_BAD_*-PA1120 expression cassette was verified by PCR using the primer pair Pser-up/Pser-down (48) and the sequence of the expression cassette was verified by sequencing.

To obtain P. aeruginosa strains exhibiting arabinose-inducible expression of the PDE PA2133, the *araC*-P*_BAD_*-PA2133 expression cassette of pENTRminiCTX2-P*_BAD_*-PA2133 was inserted into the chromosome of the P. aeruginosa strain of choice using the exact three-step protocol described above.

### Construction of P. aeruginosa deletion mutants.

To obtain P. aeruginosa *algP* deletion mutants, an allelic exchange vector, p*ΔalgP*, was initially constructed and subsequently applied to introduce a deletion in the *algP* gene as outlined in the elegant protocol developed by Hmelo and coworkers (49). Initially, two DNA fragments flanking the future *algP* deletion were PCR amplified using either the primer pair algP-up-F/algP-up-R or the primer pair algP-down-F/algP-down-R. Notably, primers had been designed so that the algP-up-F primer carried a 5′ extension containing an *attB1* site, the algP-up-R primer carried a 5′ extension complementary to the algP-down-F primer, and the algP-down-R primer carried a 5′ extension containing an *attB2* site. Then the two DNA fragments were fused by SOE PCR using the primer pair algP-up-F/algP-down-R, and the resulting PCR fragment was cloned into the gateway plasmid pDONRPEX18Gm using BP Clonase (Invitrogen) to give the allelic exchange vector pΔ*algP*. To verify the composition of the allelic-exchange insert of pΔ*algP*, plasmid DNA of pΔ*algP* was sequenced using algP-up-F and algP-down-R as sequencing primers.

Afterwards, P. aeruginosa merodiploids with the allelic-exchange vector pΔ*algP* inserted into its chromosome were created by triparental mating among DH5α/pΔ*algP* (donor), HB101/pRK600 (helper), and the P. aeruginosa strain of interest (recipient). Next, the transconjugants (merodiploids) were subjected to SacB-based counterselection by means of repeated streaking onto both LB plates supplemented with 60 μg/mL of gentamicin and no-NaCl-LB plates (no-NaCl-LB medium solidified with 15 g/L of agar (Agar Bacteriological, grade LP0011T; Oxoid, United Kingdom) supplemented with 15% sucrose). To obtain double-crossover transconjugants, sucrose selection at 30°C was repeated until the emergence of sucrose-resistant, gentamicin-sensitive colonies. Finally, PCR analysis with the *algP* flanking primer pair algP-seq-F/algP-seq-R was conducted on sucrose resistant, gentamicin-sensitive transconjugants, and double-crossover transconjugants carrying the *algP* deletion were identified and selected for further analysis.

P. aeruginosa strains carrying deletions of the genes *algD*, *algR*, *amrZ*, *algB*, *algQ*, *cysB*, *vf*r, *IHFα*, and *IHFβ* were constructed using the exact protocol described above.

### Construction of knock-in vectors.

To obtain P. aeruginosa strains encoding a mutant AlgB protein in which amino acid residue 181 has been changed from lysine to alanine (K181A mutation), an allelic-exchange knock-in vector, pENTR*algB*-K181A, was constructed as follows. Initially, two DNA fragments encoding alanine instead of lysine at amino acid residue 181 of AlgB were PCR amplified using either the primer pair algB-SDM-UpF/algB-K181A-UpR or the primer pair algB-K181A-DnF/algB-SDM-DnR. Notably, primers had been designed so that the algB-SDM-UpF primer carried a 5′ extension containing an *attB1* site, the algB-K181A-UpR primer encoded the K181A mutation, the algB-K181A-DnF primer was complementary to the algB-K181A-UpR primer, and the algB-SDM-DnR primer carried a 5′ extension containing an *attB2* site. Then, the two DNA fragments were fused by SOE PCR using the primer pair algB-SDM-UpF/algB-SDM-DnR, and the resulting PCR fragment was cloned into the gateway plasmid pDONRPEX18Gm using BP Clonase (Invitrogen) to give the allelic-exchange knock-in vector pENTR*algB*-K181A. To verify the composition of pENTR*algB*-K181A, plasmid DNA of pENTR*algB*-K181A was sequenced using primers algB-SDM-seqF and algB-SDM-seqR as sequencing primers.

In a similar manner, we created two additional allelic knock-in vectors, pENTR*algB*-R186A and pENTR*algB*-K181A-R186A (consult primer list in Table S3 in the supplemental material for details). pENTR*algB*-R186A encodes a mutant AlgB protein in which amino residue 186 has been changed from arginine to alanine (R186A mutation) and pENTR*algB*-K181A-R186A encodes a mutant AlgB protein in which amino residue 181 has been changed from lysine to alanine (K181A mutation) and amino acid residue 186 has been changed from arginine to alanine (R186A mutation).

### Construction of P. aeruginosa knock-in mutants.

Using the protocol outlined above (see “Construction of P. aeruginosa deletion mutants”), the mutant *algB*-K181A gene of pENTR*algB*-K181A was inserted into the chromosome of P. aeruginosa using triparental mating between DH5α/pENTR*algB*-K181A (donor), HB101/pRK600 (helper), and the P. aeruginosa strain of interest (recipient). To discriminate double-crossover transconjugants carrying wild-type *algB* from double-crossover transconjugants carrying the mutant *algB*-K181A gene, the *algB* alleles of 8 double-crossover transconjugants were sequenced, and among these, a double-crossover transconjugant carrying the *algB*-K181A gene was selected for further analysis.

In a similar manner, the *algB*-R186A gene of pENTR*algB*-R186A, the *algB*-K181A-R186A gene of pENTR*algB*-K181A-R186A, or the *mucA22* gene of pENTR*mucA22* was inserted into the chromosome of P. aeruginosa to give P. aeruginosa strains encoding either AlgB-R186A, AlgB-K181A-R186K, or MucA22.

### Construction of c-di-GMP monitor strains.

To create strains capable of gauging their intracellular c-di-GMP content, the reporter plasmid pCdrA-gfp (39) was electroporated into the P. aeruginosa strain of interest as outlined in the protocol reported previously by Choi and coworkers (50). Transformants of the respective strains were selected on LB plates supplemented with 60 μg/mL of gentamicin, and from these plates, one green fluorescent protein (GFP)-positive transformant of each strain was picked for c-di-GMP level assessments.

### Construction of pET28b-MBP.

The *mbp* gene was cut out of plasmid pMAL-C2x (51) via the NdeI and EcoRI sites and was inserted into the pET28b vector (Novagen) via the same restriction sites to make the pET28b-MBP plasmid.

### Construction of AlgB, AlgR, and AmrZ production strains.

To acquire high-purity protein stocks of either AlgB, AlgR, or AmrZ suitable for ligand binding assays, expression vectors for synthesis of either AlgB, AlgR, or AmrZ protein were created as follows. Initially, a DNA fragment bearing the *algB* gene of P. aeruginosa flanked by a 5′ BamHI-site and a 3′ EcoRI site was PCR amplified from chromosomal DNA of P. aeruginosa using the primer pair GST-algB-F/GST-algB-R (Table S3 in supplemental data). The resulting PCR fragment was then digested with BamHI and EcoRI and cloned into the corresponding sites of digested pGEX-6P-2 (GE Healthcare) to give the AlgB expression vector pGST-AlgB. To verify that pGST-AlgB carried the expected in-frame fusion between *gst* and *algB* that would enable synthesis of full-length AlgB of high purity, pGST-AlgB was sequenced using the primer pair pGEX-seqF and pGEX-seqR. Finally, pGST-AlgB was electroporated into the protein expression-optimized E. coli strain Rosetta DE3/pLysS to generate an efficient AlgB production strain.

In a similar way, we PCR amplified DNA fragments flanked by a SalI site and a NotI site and carrying either *algR* or *amrZ* from chromosomal DNA of P. aeruginosa using either the primer pair AlgR-SalI-F/AlgR-NotI-R or the primer pair AmrZ-SalI-F/AlgR-NotI-R. The 2 resulting PCR fragments were then digested with SalI and NotI and cloned into the SalI site and the NotI site of pET28b-MBP to give the AlgR expression vector pET28b-MBP-AlgR and the AmrZ expression vector pET28b-MBP-AmrZ, respectively. To verify that pET28b-MBP-AlgR carried the expected in-frame fusion between *mbp* and *algR* that would enable synthesis of full-length AlgR of high purity, pET28b-MBP-AlgR was sequenced using primer pET28b-seqF and primer pET28b-seqR as sequencing primers. Likewise, to verify that pET28b-MBP-AmrZ carried the expected in-frame fusion between *mbp* and *amrZ* that would enable synthesis of full-length AmrZ of high purity, pET28b-MBP-AmrZ was sequenced using primer pET28b-seqF and primer pET28b-seqR as sequencing primers. Finally, pET28b-MBP-AlgR and pET28b-MBP-AmrZ were electroporated into the protein expression-optimized E. coli strain Rosetta DE3/pLysS to generate an efficient AlgR production strain and an efficient AmrZ production strain.

### Purification of recombinant GST-AlgB, His-AlgR, and His-AmrZ proteins.

For purification of the AlgB protein, isopropyl-β-d-thiogalactopyranoside (IPTG; 100 μM) was used to induce the E. coli/pGST-algB expression strain in a late-log-phase culture (optical density at 600 nm [OD_600_] from 0.8 to 1.0) at 18°C and 90 rpm for 12 h. The AlgB expression culture was harvested by centrifugation (5,000 × *g*, 20 min, 4°C, with precooling of everything on ice) and resuspended in buffer containing 96% (vol/vol) HN150G buffer (50 mM HEPES [pH 7.5], 150 mM NaCl, and 10% glycerol), 4% (vol/vol) Triton X-100, 0.5% (wt/vol) 3-[(3-cholamidopropyl)-dimethylammonio]-1-propanesulfonate (CHAPS), 2× Roche Complete Ultra Tabs, and 5 mM dithiothreitol (DTT). The cell lysate was centrifuged at 12,000 rpm and 4°C for 1 h, and the supernatant was loaded onto an affinity chromatography column containing 1 mL of affinity resin glutathione Sepharose 4 Fast Flow (GE Healthcare). Eighty microliters (160 U) of PreScission protease (Sigma-Aldrich) together with 920 μL of pH 7.0 cleavage buffer (50 mM Tris-HCl, 150 mM NaCl, 1 mM EDTA, and 1 mM DTT) was applied to do on-column cleavage at 4°C for 8 h. Then 3 mL of cleavage buffer was washed through the column and the cleaved AlgB protein was gathered in pH 8.0 elution buffer (50 mM Tris-HCl, 10 mM reduced glutathione). Purification of His-AlgR and His-AmrZ was performed as described previously (34). Protein concentrations were assessed using a Bradford assay (Bio-Rad), and the proteins were stored at 4°C before performing the DRaCALA assays the following day.

### Assessment of functionality of the *algD-gfp* reporter strains.

To verify the functionality of the alginate transcription reporter constructed in this study, the GFP fluorescence output from single colonies of various P. aeruginosa *algD-gfp* reporter strains was visualized using fluorescence microscopy. At first, chromosomally Tn*7*::*algD-gfp*-tagged strains of either wild-type, *mucA22*, Δ*algD*, or Δ*algD mucA22* PAO1 were streaked onto LB plates and incubated at 37°C. Following 20 h of cultivation, bright-field images and epifluorescence images of single colonies of each of the 4 *algD-gfp* monitor strains were recorded using a Zeiss LSM710 confocal microscope (×5 objective, no. 0.2; 488-nm excitation and emission band of 495 to 530 nm). The acquired images were processed by Imaris 9.5 software (Bitplane; Oxford Instruments, United Kingdom) as described by Kragh et al. (52).

### Gauging transcription of the alginate biosynthesis operon by means of *algD-gfp* monitor strains.

To evaluate the impact of the intracellular concentration of c-di-GMP on the transcription of the alginate biosynthesis operon (PA3540-PA3548), the GFP fluorescence arising from *algD*-*gfp* reporter strains encoding arabinose-inducible synthesis of either PA2133 (PDE) or PA1120 (DGC) was determined using a 96-well microtiter setup. Initially, 18-h-old cultures of either OJ108 (PAO1 Δ*pel* Δ*psl* Δ*alg mucA22* Tn*7*::*algD-gfp* CTX::*araC*-P*_BAD_*-PA1120) or OJ109 (PAO1 Δ*pel* Δ*psl* Δ*alg mucA22* Tn*7*::*algD-gfp* CTX::*araC*-P*_BAD_*-PA2133) (propagated in MTB medium) was diluted into either MBT medium or MTB medium supplemented with 0.2% arabinose to obtain starter cultures exhibiting an OD_600_ value of 0.025. Then 200-μL aliquots of the 4 different starter cultures were distributed into the wells of a 96-well microtiter plate (black plate; Nunc). The resulting microtiter plate was sealed with a lid and incubated in a Tecan reader (Infinite F200 PRO) at 37°C and 440 rpm, and corresponding values of cell density (OD_600_) and GFP fluorescence (fluorescence units [FU]) were measured every 20 min for 18 h. Finally, to visualize the impact of c-di-GMP content on transcription of the alginate biosynthesis operon (PA3540-PA3548), a bar diagram displaying the maximum GFP values observed for strain OJ108 cultivated in the absence of arabinose (DGC) and in the presence of arabinose (DGC+) and the maximum GFP values observed for strain OJ109 cultivated in the absence of arabinose (PDE) and in the presence of arabinose (PDE+) was constructed (Fig. 2). In Fig. 2, the maximum GFP values are the averages of 5 independent wells (technical replicates) across 3 independent experiments (biological replicates).

In a similar way, we also examined how mutants of the genes *algP*, *algQ*, *IHFα*, *IHFβ*, *vfr*, *cysB*, *amrZ*, *algR*, and *algB* affected the transcription of the alginate biosynthesis operon (PA3540-PA3548) at either reduced, or increased concentrations of intracellular c-di-GMP. Initially, starter cultures of OJ108, OJ109, ZWKO11, ZWKO12, ZWKO13, ZWKO14, ZWKO15, ZWKO16, ZWKO17, ZWKO18, ZWKO19, ZWKO20, ZWKO21, ZWKO22, ZWKO23, ZWKO24, ZWKO25, ZWKO26, ZWKO27, and ZWKO28 diluted to an OD_600_ value of 0.025 in MTB medium supplemented with 0.2% arabinose were created as outlined above. Then 200-μL aliquots of all the different starter cultures were distributed into 96-well microtiter plates (black plate; Nunc). The resulting microtiter plates were sealed with a lid and incubated in a Tecan reader (Infinite F200 PRO) at 37°C and 440 rpm, and corresponding values of cell density (OD_600_) and GFP fluorescence (FU) were measured every 20 min for 18 h. Finally, to visualize how the various mutants affected the transcription of the alginate biosynthesis operon (PA3540-PA3548) at either reduced or increased c-di-GMP concentrations, a bar diagram displaying the maximum GFP values measured in arabinose-treated cultures of each mutant strain (ZWKO11 to ZWKO28) was constructed (Fig. 3). In Fig. 3, the maximum GFP values are the averages of 5 independent wells (technical replicates) across 3 independent experiments (biological replicates).

Using the exact same procedure as described above, we also examined how the *algB*-K181A, *algB*-R186A, *algB*-K181A-R186A, and Δ*algB* mutated strains affected the transcription of the alginate biosynthesis operon (PA3540-PA3548) at either reduced or increased concentrations of intracellular c-di-GMP. To clarify if any of the respective mutants influenced the transcription of the alginate synthesis operon (PA3540-PA3548), a bar plot displaying the maximum GFP values observed in arabinose-treated cultures of OJ108, OJ109, ZW07 to ZW12, ZWKO27, ZWKO28 and MTR856, MTR857, ZW01 to ZW06, ZWKO33, and ZWKO34 was created (Fig. 5). In Fig. 5, the maximum GFP values are the averages of 3 independent wells (technical replicates) across 3 independent experiments (biological replicates).

### Gauging the intracellular c-di-GMP content by means of pCdrA-gfp monitor strains.

To estimate the c-di-GMP content experienced by the *algD*-*gfp* monitor strains OJ108 and OJ109 and by the *algR*, *algB*, and *amrZ* mutants of OJ108 and OJ109, the GFP fluorescence arising from the pCdrA-gfp reporter plasmid of strains ZW28 to ZW35 and MTR856, MTR857, and ZWKO29 to ZWKO34 were determined using a 96-well microtiter setup. Initially, 18-h-old cultures of ZW28 to ZW35 and MTR856, MTR857, and ZWKO29 to ZWKO34 were diluted into MTB medium supplemented with 60 μg/mL of gentamicin and 0.2% arabinose to create starter cultures exhibiting an OD_600_ value of 0.025. Then 200-μL aliquots of the 16 different starter cultures were distributed into 96-well microtiter plates (black plate; Nunc). The resulting microtiter plates were sealed with a lid and incubated at 37°C and 440 rpm in a Tecan reader (Infinite F200 PRO), and corresponding values of cell density (OD_600_) and GFP fluorescence (FU) were measured every 20 min for 18 h. Finally, to visualize the c-di-GMP content experienced by the *algD*-*gfp* monitor strains OJ108 and OJ109 and by the *algR*, *algB*, and *amrZ* mutants of OJ108 and OJ109 under the growth conditions applied in this study, a bar plot displaying the maximum GFP values measured in arabinose-treated cultures of each test strain (ZW28 to ZW35 and MTR856, MTR857, and ZWKO29 to ZWKO34) was constructed (Fig. 6). In Fig. 6, the maximum GFP values are the averages of 3 independent wells (technical replicates) across 3 independent experiments (biological replicates).

### DRaCALA binding assay.

The DRaCALA c-di-GMP binding assay of purified AlgB, AmrZ, and AlgR protein and the preparation of the whole-cell lysates were performed as previously described by Schicketanz et al. (34). The whole-cell lysates containing the E. coli IlvH protein overexpressed from the plasmid pCA24N-ilvH were used as the positive control, since the IlvH protein is known to bind c-di-GMP (35), and a whole-cell lysate with an empty vector pCA24N (36) was used as the negative control. The radioactive c-di-GMP was synthesized from ^32^P-α-GTP (Perkin Elmer) via the purified His-YdeH protein (53).

### CFU counts and clumping captured by confocal laser scanning microscopy.

During growth experiments with P. aeruginosa strains encoding arabinose-inducible expression of the DGC PA1120, we constantly observed that cultures of these strains gave rise to significantly lower cell densities if arabinose was added to the growth media, although these strains were unable to synthesize any of the clumping-inducing exopolysaccharides Pel, Psl, and alginate. So in order to clarify whether the arabinose-dependent differences in OD_600_ values of strain OJ108 reflected differences in growth yield (CFU) or reflected differences in aggregate formation (clumping), the following experiment was conducted. Initially, an 18-h-old culture of strain OJ108 was diluted to an OD_600_ value of 0.025 in either MTB medium or MTB medium supplemented with 0.2% arabinose (wt/vol), and the resulting starter cultures were distributed into a 96-well microtiter plate as 200-μL aliquots. The microtiter plate was sealed with a lid and incubated in a Tecan reader (Infinite F200 PRO) at 37°C and 440 rpm, and OD values were measured every 20 min for 18 h. Following 18 h of cultivation, 3 × 100-μL aliquots of either untreated or arabinose-treated cultures were sampled. Half of the samples were degassed for 5 min and subsequently sonicated for 5 min at 42 kHz (Bransonic; Ultrasonic 2510), while the other half of the samples were dyed with SYTO 9 green fluorescent nucleic acid stain (Thermo Fisher, 3 mM in saline) as described by Kragh et al. (54), to evaluate the degree of aggregation. The sonicated culture samples were appropriately diluted and spread onto LB plates, and following 24 h of incubation at 37°C, the colonies that had emerged on the plates were enumerated. Then the number of bacteria (CFU) present in each sonicated culture sample was calculated and displayed in bar plot (Fig. S2A in the supplemental material). To evaluate the degree of bacterial aggregate formation in untreated and arabinose-treated cultures of strain OJ108, the SYTO 9-stained samples of the respective cultures were applied to Ibidi IV μ-slide microscopy slides (Ibidi, Germany) and imaged as z-stacks using an LSM880 confocal microscope (equipped with a 63× 1.4 oil objective and 488-nm excitation laser and a emission band of 495 to 530 nm). The acquired images (Fig. S2B in the supplemental material) were analyzed/processed by Imaris 9.5 software (Bitplane; Oxford Instruments, United Kingdom) (54).

### Statistical analysis.

The data presented in Fig. 3, 5, and 6 were analyzed using two-way analysis of variance (ANOVA) with Tukey’s multiple-comparison test. The data presented in Fig. 2 were analyzed using ordinary one-way ANOVA with Šídák’s multiple-comparison test. *P* values less than 0.05 indicated significant differences.
