# Supplementary material for: Transcription of the Alginate Operon in Pseudomonas aeruginosa Is Regulated by c-di-GMP
Source: Microbiol Spectr. 2022 Jul 11;10(4):e00675-22. doi: 10.1128/spectrum.00675-22 (PMC9431422; doi:10.1128/spectrum.00675-22)
Supplement: Supplemental file 1 — Supplemental material. Download spectrum.00675-22-s0001.pdf, PDF file, 0.8 MB [file spectrum.00675-22-s0001.pdf]

Supplementary Materials

Transcription of the alginate operon in *Pseudomonas aeruginosa* is regulated by c-di-GMP

Ziwei Liang[#,a], Morten Rybtke[#,a], Kasper Nørskov Kragh[a], Owen Johnson[a], Muriel Schicketanz[b], Yong Everett Zhang[b], Jens Bo Andersen[¤,a], Tim Tolker-Nielsen[*,a]

[a] Costerton Biofilm Center, Department of Immunology and Microbiology, Faculty of Health and Medical Sciences, University of Copenhagen, Copenhagen, Denmark

[b] Department of Biology, Copenhagen Biocenter, University of Copenhagen, Copenhagen, Denmark

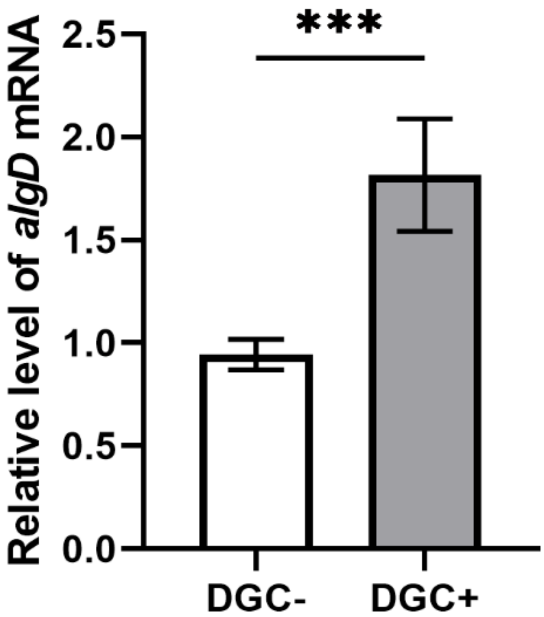

Fig. S1. qRT-PCR analysis suggests that transcription of the alginate operon in *P. aeruginosa* is positively regulated by c-di-GMP. Quantities of *algD* mRNA was determined in late-log cultures of *P. aeruginosa* mucA22CTX::araC-PBAD-PA1120 without arabinose (DGC-) or with arabinose (DGC+) using qRT-PCR as described by Andersen et al. (1). Data were analyzed by the 2-ΔΔCt method using *rpoD* and *oprL* as endogenous control genes. Mean and standard deviations are based on 2 biological replicates, each with 3 technical replicates. Unpaired t-test was carried out to assess the significance level of the difference between the values; *** $p<0.001$.

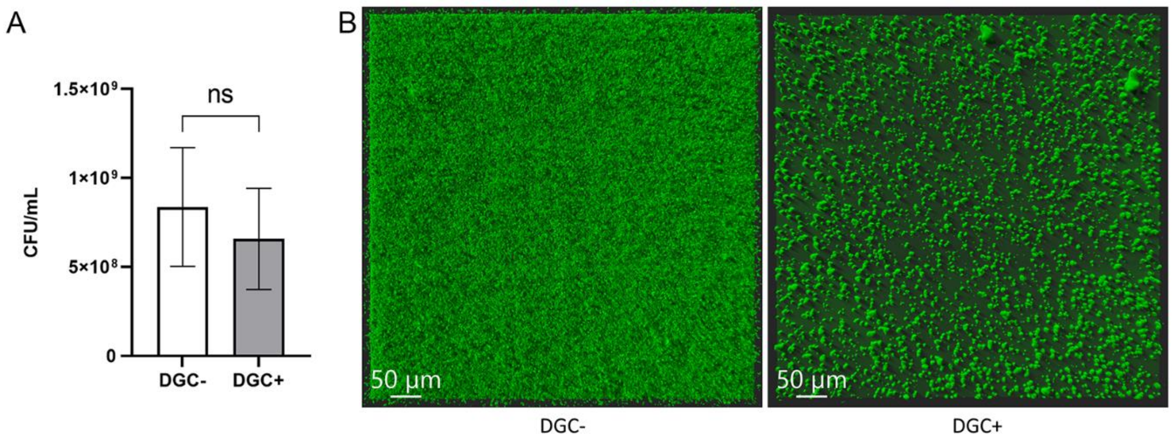

Fig. S2. Over-expression of PA1120 in *P. aeruginosa* causes cell aggregation independent of matrix exopolysaccharides. (**A**) Colony forming units (CFU) of late log cultures of *ΔpelΔpslΔalgDmucA22*Tn7::*algD-gfp*CTX::*araC*-P_{BAD}-PA1120 without arabinose (DGC-) or with arabinose (DGC+). Mean and standard deviation (bars) of 9 replicates are shown. Unpaired t-test was carried out to assess the significance level of the difference between the values; ns indicates no significant difference. (**B**) Confocal laser scanning microscopy images of samples from late log cultures of *ΔpelΔpslΔalgDmucA22*Tn7::*algD-gfp*CTX::*araC*-P_{BAD}-PA1120 without arabinose (DGC-) or with arabinose (DGC+). Size bars are 50 μm.

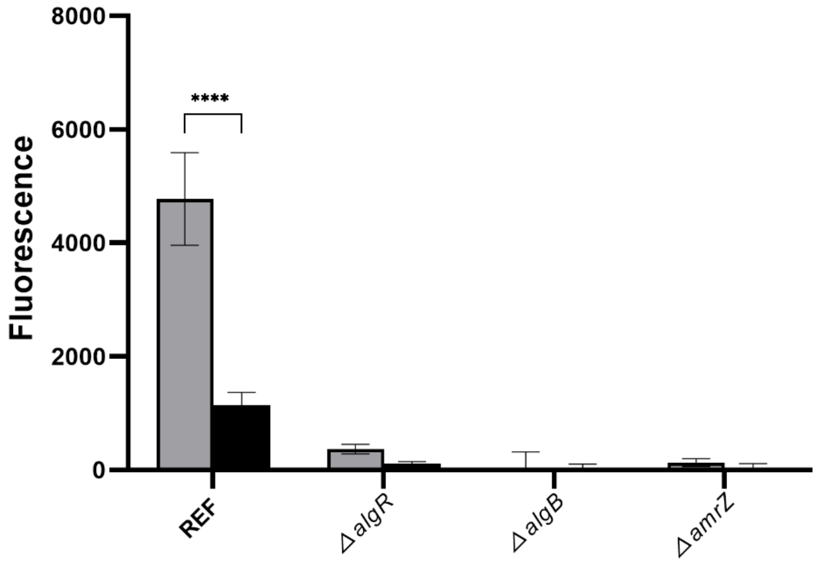

Fig. S3. The AmrZ, AlgR and AlgB proteins are absolutely required for transcription of the alginate operon in *P. aeruginosa*. The two bars labelled REF shows fluorescence of late-log cultures of *ΔpelΔpslΔalgDmucA22*Tn7::*algD-gfp*CTX::*araC*-P_{BAD}-PA1120 with arabinose (grey bar) and *ΔpelΔpslΔalgDmucA22* Tn7::*algD-gfp*CTX::*araC*-P_{BAD}-PA2133 with arabinose (black bar). The following bars shows fluorescence of late-log cultures of the same two background strains with additional gene deletions as indicated. Fluorescence of cultures of the corresponding strains without the Tn7::*algD-gfp* fusion are withdrawn. Mean and standard deviation (bars) of 9 replicates are shown. One-way ANOVA analysis was carried out to assess the significance level of the difference between the values; **** p<0.0001.

Table S1. Bacterial strains used in the study.

Strain	Relevant genotype and/or characteristics	Reference/source
P. aeruginosa strains		
PAO1	*P. aeruginosa* reference strain	(2)
MTR838	PAO1(*mucA22*)	This study
MTR822	PAO1(*ΔalgD*)	This study
MTR841	PAO1(*ΔalgD mucA22*)	This study
MTR152	PAO1(*Δpel Δpsl*)	(3)
MTR837	PAO1(*Δpel Δpsl ΔalgD*)	This study
MTR851	PAO1(*Δpel Δpsl Δalg mucA22*)	This study
MTR856	PAO1(*Δpel Δpsl ΔalgD mucA22* CTX::*araC*-P_{BAD}-PA1120)	This study
MTR857	PAO1(*Δpel Δpsl ΔalgD mucA22* CTX::*araC*-P_{BAD}-PA2133)	This study
ZWKO29	PAO1(*ΔalgR Δpel Δpsl ΔalgD mucA22* CTX::*araC*-P_{BAD}-PA1120)	This study
ZWKO30	PAO1(*ΔalgR Δpel Δpsl ΔalgD mucA22* CTX::*araC*-P_{BAD}-PA2133)	This study
ZWKO31	PAO1(*ΔamrZ Δpel Δpsl ΔalgD mucA22* CTX::*araC*-P_{BAD}-PA1120)	This study
ZWKO32	PAO1(*ΔamrZ Δpel Δpsl ΔalgD mucA22* CTX::*araC*-P_{BAD}-PA2133)	This study
ZWKO33	PAO1(*ΔalgB Δpel Δpsl ΔalgD mucA22* CTX::*araC*-P_{BAD}-PA1120)	This study
ZWKO34	PAO1(*ΔalgB Δpel Δpsl ΔalgD mucA22* CTX::*araC*-P_{BAD}-PA2133)	This study
MTR942	PAO1(*Δpel Δpsl ΔalgD mucA22*) encoding AlgB-K181A	This study
MTR944	PAO1(*Δpel Δpsl ΔalgD mucA22*) encoding AlgB-R186A	This study
MTR946	PAO1(*Δpel Δpsl ΔalgD mucA22*) encoding AlgB-K181A-R186A	This study
ZW01	PAO1(*Δpel Δpsl ΔalgD mucA22* CTX::*araC*-P_{BAD}-PA1120) encoding AlgB-K181A	This study
ZW02	PAO1(*Δpel Δpsl ΔalgD mucA22* CTX::*araC*-P_{BAD}-PA1120) encoding AlgB-R186A	This study
ZW03	PAO1(*Δpel Δpsl ΔalgD mucA22* CTX::*araC*-P_{BAD}-PA1120) encoding AlgB-K181A-R186A	This study
ZW04	PAO1(*Δpel Δpsl ΔalgD mucA22* CTX::*araC*-P_{BAD}-PA2133) encoding AlgB-K181A	This study
ZW05	PAO1(*Δpel Δpsl ΔalgD mucA22* CTX::*araC*-P_{BAD}-PA2133) encoding AlgB-R186A	This study
ZW06	PAO1(*Δpel Δpsl ΔalgD mucA22* CTX::*araC*-P_{BAD}-PA2133) encoding AlgB-K181A-R186A	This study

ZW36	PAO1(Tn7::*algD-gfp*)	This study
ZW37	PAO1(*mucA22* Tn7::*algD-gfp*)	This study
MTR889	PAO1(*ΔalgD* Tn7::*algD-gfp*)	This study
MTR890	PAO1(*ΔalgD mucA22* Tn7::*algD-gfp*)	This study
OJ107	PAO1(*Δpel Δpsl Δalg mucA22* Tn7::*algD-gfp*)	This study
OJ108	PAO1(*Δpel Δpsl ΔalgD mucA22* Tn7::*algD-gfp* CTX::*araC*-P_{BAD}-PA1120)	This study
OJ109	PAO1(*Δpel Δpsl ΔalgD mucA22* Tn7::*algD-gfp* CTX::*araC*-P_{BAD}-PA2133)	This study
ZWKO11	PAO1(*ΔalgP Δpel Δpsl ΔalgD mucA22* Tn7::*algD-gfp* CTX::*araC*-P_{BAD}-PA1120)	This study
ZWKO12	PAO1(*ΔalgP Δpel Δpsl ΔalgD mucA22* Tn7::*algD-gfp* CTX::*araC*-P_{BAD}-PA2133)	This study
ZWKO13	PAO1(*ΔalgR Δpel Δpsl ΔalgD mucA22* Tn7::*algD-gfp* CTX::*araC*-P_{BAD}-PA1120)	This study
ZWKO14	PAO1(*ΔalgR Δpel Δpsl ΔalgD mucA22* Tn7::*algD-gfp* CTX::*araC*-P_{BAD}-PA2133)	This study
ZWKO15	PAO1(*ΔalgQ Δpel Δpsl ΔalgD mucA22* Tn7::*algD-gfp* CTX::*araC*-P_{BAD}-PA1120)	This study
ZWKO16	PAO1(*ΔalgQ Δpel Δpsl ΔalgD mucA22* Tn7::*algD-gfp* CTX::*araC*-P_{BAD}-PA2133)	This study
ZWKO17	PAO1(*ΔamrZ Δpel Δpsl ΔalgD mucA22* Tn7::*algD-gfp* CTX::*araC*-P_{BAD}-PA1120)	This study
ZWKO18	PAO1(*ΔamrZ Δpel Δpsl ΔalgD mucA22* Tn7::*algD-gfp* CTX::*araC*-P_{BAD}-PA2133)	This study
ZWKO19	PAO1(*ΔcysB Δpel Δpsl ΔalgD mucA22* Tn7::*algD-gfp* CTX::*araC*-P_{BAD}-PA1120)	This study
ZWKO20	PAO1(*ΔcysB Δpel Δpsl ΔalgD mucA22* Tn7::*algD-gfp* CTX::*araC*-P_{BAD}-PA2133)	This study
ZWKO21	PAO1(*Δvrf Δpel Δpsl ΔalgD mucA22* Tn7::*algD-gfp* CTX::*araC*-P_{BAD}-PA1120)	This study
ZWKO22	PAO1(*Δvrf Δpel Δpsl ΔalgD mucA22* Tn7::*algD-gfp* CTX::*araC*-P_{BAD}-PA2133)	This study
ZWKO23	PAO1(*ΔIHFα Δpel Δpsl ΔalgD mucA22* Tn7::*algD-gfp* CTX::*araC*-P_{BAD}-PA1120)	This study
ZWKO24	PAO1(*ΔIHFα Δpel Δpsl ΔalgD mucA22* Tn7::*algD-gfp* CTX::*araC*-P_{BAD}-PA2133)	This study
ZWKO25	PAO1(*ΔIHFβ Δpel Δpsl ΔalgD mucA22* Tn7::*algD-gfp* CTX::*araC*-P_{BAD}-PA1120)	This study
ZWKO26	PAO1(*ΔIHFβ Δpel Δpsl ΔalgD mucA22* Tn7::*algD-gfp* CTX::*araC*-P_{BAD}-PA2133)	This study
ZWKO27	PAO1(*ΔalgB Δpel Δpsl ΔalgD mucA22* Tn7::*algD-gfp* CTX::*araC*-P_{BAD}-PA1120)	This study
ZWKO28	PAO1(*ΔalgB Δpel Δpsl ΔalgD mucA22* Tn7::*algD-gfp* CTX::*araC*-P_{BAD}-PA2133)	This study
ZW07	PAO1(*Δpel Δpsl ΔalgD mucA22* CTX::*araC*-P_{BAD}-PA1120 Tn7::algD-*gfp*) encoding AlgB-K181A, Gm^r	This study
ZW08	PAO1(*Δpel Δpsl ΔalgD mucA22* CTX::*araC*-P_{BAD}-PA1120 Tn7::algD-*gfp*) encoding AlgB-R186A, Gm^r	This study

ZW09	PAO1(*Δpel Δpsl ΔalgD mucA22* CTX::*araC*-P_{BAD}-PA1120 Tn7::algD-*gfp*) encoding AlgB-K181A-R186A, Gm^r	This study
ZW10	PAO1(*Δpel Δpsl ΔalgD mucA22* CTX::*araC*-P_{BAD}-PA2133 Tn7::algD-*gfp*) encoding AlgB-K181A, Gm^r	This study
ZW11	PAO1(*Δpel Δpsl ΔalgD mucA22* CTX::*araC*-P_{BAD}-PA2133 Tn7::algD-*gfp*) encoding AlgB-R186A, Gm^r	This study
ZW12	PAO1(*Δpel Δpsl ΔalgD mucA22* CTX::*araC*-P_{BAD}-PA2133 Tn7::algD-*gfp*) encoding AlgB-K181A-R186A, Gm^r	This study
ZW28	PAO1(*Δpel Δpsl ΔalgD mucA22* CTX::*araC*-P_{BAD}-PA1120) carrying pCdrA-gfp, Gm^r	This study
ZW29	PAO1(*Δpel Δpsl ΔalgD mucA22* CTX::*araC*-P_{BAD}-PA2133) carrying pCdrA-gfp, Gm^r	This study
ZW30	PAO1(*ΔalgR Δpel Δpsl ΔalgD mucA22* CTX::*araC*-P_{BAD}-PA1120) carrying pCdrA-*gfp*, Gm^r	This study
ZW31	PAO1(*ΔalgR Δpel Δpsl ΔalgD mucA22* CTX::*araC*-P_{BAD}-PA2133) carrying pCdrA-*gfp*, Gm^r	This study
ZW32	PAO1(*ΔamrZ Δpel Δpsl ΔalgD mucA22* CTX::*araC*-P_{BAD}-PA1120) carrying pCdrA-*gfp*, Gm^r	This study
ZW33	PAO1(*ΔamrZ Δpel Δpsl ΔalgD mucA22* CTX::*araC*-P_{BAD}-PA2133) carrying pCdrA-*gfp*, Gm^r	This study
ZW34	PAO1(*ΔalgB Δpel Δpsl ΔalgD mucA22* CTX::*araC*-P_{BAD}-PA1120) carrying pCdrA-*gfp*, Gm^r	This study
ZW35	PAO1(*ΔalgB Δpel Δpsl ΔalgD mucA22* CTX::*araC*-P_{BAD}-PA2133) carrying pCdrA-*gfp*, Gm^r	This study
ZW38	PAO1(*mucA22* CTX::*araC*-P_{BAD}-PA1120)	This study
E. coli strains		
DH5α	F^- *endA1 glnV44 thi-1 recA1 relA1 gyrA96 deoR nupG purB20 Φ80dlacZΔM15 Δ(lacZYA-argF) U169 hsdR17* $(r_k^- m_k^+)$ $λ^-$	lab collection
HB101	*recA thi pro leu hsdRM1* Sm^r	(4)
Rosetta DE3 pLysS	F^- *ompT hsdS*$_B$(r_B^- m_B^-) *gal dcm* DE3 pLysSRARE, Cm^r	Novagen

Table S2. Plasmids used in the study.

Plasmids	Relevant genotype and/or characteristics	Reference or source
pDONRPEX18Gm	Gateway donor, Gm^r	(5)
pFlp2	Source of Flp2 recombinase, Amp^r	(5)
pUX-BF13	Tn7 transposase-encoding plasmid	(6)
pRK600	Mobilization plasmid, Cm^r	(4)
pUC18-miniTn7T-Gm	Tn7 transposon delivery vector, Gm^r	(7)

Fluorescent monitors:		
pTn7::*algD-gfp*	Tn7 transposon delivery vector encoding the *alg*D promoter-*gfp* fusion of p*alg*D-*gfp*, Gm^r	This study
pCdrA-gfp	c-di-GMP monitor plasmid, Gm^r	(3)
Knock-in/-out vectors:		
pENTRmucA22	Knock-in vector creating the *mucA22* mutant gene, Gm^r	This study
pENTRalgB-K181A	Knock-in vector creating a K181A amino acid change in AlgB, Gm^r	This study
pENTRalgB-R186A	Knock-in vector creating a R186A amino acid change in AlgB, Gm^r	This study
pENTRalgB-K181A-R186A	Knock-in vector creating a K181A and a R186A amino acid change in AlgB, Gm^r	This study
pΔ*algD*	pDONRPEX18Gm based *algD* knockout vector, Gm^r	(8)
pΔ*algP*	pDONRPEX18Gm based *algP* knockout vector, Gm^r	This study
pΔ*algR*	pDONRPEX18Gm based *algR* knockout vector, Gm^r	This study
pΔ*algQ*	pDONRPEX18Gm based *algQ* knockout vector, Gm^r	This study
pΔ*amrZ*	pDONRPEX18Gm based *amrZ* knockout vector, Gm^r	This study
pΔ*cysB*	pDONRPEX18Gm based *cysB* knockout vector, Gm^r	This study
pJJH107	pEX18Gm based *vfr* knockout vector, Gm^r	(9)
pΔ*IHFα*	pDONRPEX18Gm based *IHFα* knockout vector, Gm^r	This study
pΔ*IHFβ*	pDONRPEX18Gm based *IHFβ* knockout vector, Gm^r	This study
pΔ*algB*	pDONRPEX18Gm based *algB* knockout vector, Gm^r	This study
Protein expression vectors:		
pGST-*algB*	GST-tagged AlgB expression vector, Amp^r	This study
pET28b-MBP-*algR*	His-tagged AlgR expression vector, Kana^r	This study
pET28b-MBP-*amrZ*	His-tagged AmrZ expression vector, Kana^r	This study

c-di-GMP manipulation vectors:		
pENTRminiCTX2-P_{BAD}-PA1120	Arabinose inducible *araC*-P_{BAD}-PA1120 fusion of pJN1120 inserted into the Gateway compapatible integration vector pDONRminiCTX2.	(3)
pENTRminiCTX2-P_{BAD}-PA2133	Arabinose inducible *araC*-P_{BAD}-PA2133 fusion of pJN2133 inserted into the Gateway compapatible integration vector pDONRminiCTX2.	H. Almblad and J.J Harrison

Table S3. Primers used in the study.

Primer name	Nucleotide sequence 5'-3'	Reference or source
algB-SDM_UpF	GGGGACAAGTTTGTACAAAAAAGCAGGCTCACTAACCCAG AAGCCGAATGG	This study
algB-K181A_UpR	TGCCAGTTCGCCGGCGCCGGAGCCGGATTCG	This study
algB-K181A_DnF	TCCGGCTCCGGCGCCGGCGAACTGGCACG	This study
algB-R186A_UpR	GTGTGGATGGCGGCTGCCAGTTCGCCCTTG	This study
algB-R186A_DnF	GGCGAACTGGCAGCCGCCATCCACACCTGG	This study
algB-SDM_DnR	GGGGACCACTTTGTACAAGAAAGCTGGGTATTCCTGGTTG CAGATGATGC	This study
algB-SDM_seqF	CCCAAGAACAGACATCGGCAG	This study
algB-SDM_seqR	CTTTCTCCAGGTCTTCCAGGC	This study
algB-K181A_cPCR	GAATCCGGCTCCGGCAAG	This study
algB-R186A_cPCR	CAAGGGCGAACTGGCACG	This study
algP-seq-F	CGAAGGAATCACCACTCGCCA	This study
algP-seq-R	CAACAGCGCGAACAAGCTGGA	This study
algP-up-F	GGGGACAAGTTTGTACAAAAAAGCAGGCTCAGTAACGGA CGTGTACCTGGGTT	This study
algP-up-R	CTTTGCAGACAGCGCCTTAGGACTTGTTGGCCGACATGAC GT	This study
algP-down-F	TCCTAAGGCGCTGTCTGCAAAG	This study
algP-down-R	GGGGACCACTTTGTACAAGAAAGCTGGGTAGATTGAGGAG TCGGATCATGGC	This study
algQ-seq-F	CGCTGTACCTGGAACATGTGGTT	This study

algQ-seq-R	CCGATTCGTTTGGAGAAACCGC	This study
algQ-up-F	GGGGACAAGTTTGTACAAAAAAGCAGGCTCAGTATCTACTCCGTGCTGGTGTTC	This study
algQ-up-R	GACTCGCCGGTGAATCAGACGAGCATGTTGTCCTCGCTTCC	This study
algQ-down-F	GTCTGATTCACCGGCGAGTC	This study
algQ-down-R	GGGGACCACTTTGTACAAGAAAGCTGGGTAGCTATCCGAGCTACTTCTCGGA	This study
algR-seq-F	TCAATCTGTATCTGCGGCAC	This study
algR-seq-R	TACGGCATTCGATGCCGAC	This study
algR-up-F	GGGGACAAGTTTGTACAAAAAAGCAGGCTCATTCAACAGCCTGAACAGCATCG	This study
algR-up-R	CCGTCAGAGCTGATGCATCAGATTCATAAGCTCAGGCTTCCTGC	This study
algR-down-F	CTGATGCATCAGCTCTGACGG	This study
algR-down-R	GGGGACCACTTTGTACAAGAAAGCTGGGTACAGGAAACGGATCTGCAGGTC	This study
armZ-seq-F	TTCCAGGGCCTGGCGCTTCT	This study
armZ-seq-R	CACGTGCGCAGATGAAGACGC	This study
armZ-up-F	GGGGACAAGTTTGTACAAAAAAGCAGGCTCATAACCGATGTCGGCGCGCAG	This study
armZ-up-R	TACGCGTGGGCTTCGGCGCTCACAGTGGGCGCATACATTGAACCTG	This study
armZ-down-F	TGAGCGCCGAAGCCCACGCGTA	This study
armZ-down-R	GGGGACCACTTTGTACAAGAAAGCTGGGTAGGGGAGACCAGCCTACGATTCGCC	This study
cysB-seq-F	CAGGGAGAAAAGGGATTTAC	This study
cysB-seq-R	TGCTGGAAATCGACGGTG	This study
cysB-up-F	GGGGACAAGTTTGTACAAAAAAGCAGGCTCAGCTCCCTATGTCCTGCAAC	This study
cysB-up-R	CGATCAGTAGACCGGCAGTTCAAGCTTCATGGAGATTCCTC	This study
cysB-down-F	GAACTGCCGGTCTACTGATCG	This study
cysB-down-R	GGGGACCACTTTGTACAAGAAAGCTGGGTAGCTATATCAGCATCGGCTG	This study
IHFα-seq-F	GTCCGCCTGTTTGAAAGTG	This study
IHFα-seq-R	GGAATGGAATCGAGATCAAC	This study
IHFα-up-F	GGGGACAAGTTTGTACAAAAAAGCAGGCTCATGGACTTCTTCGACGCCAAG	This study
IHFα-up-R	CAGCTCGTCGTTATGACTTAGCCCCCATACGCTATTTC	This study
IHFα-down-F	AAGTCATAACGACGAGCTG	This study
IHFα-down-R	GGGGACCACTTTGTACAAGAAAGCTGGGTACTACTAACTAGGCAGCGACAC	This study

IHF β-seq-F	TATCTCCGGTACCATCAAGTC	This study
IHF β -seq-R	ATCGAAAAGTCCAGCTACTC	This study
IHF β-up-F	GGGGACAAGTTTGTACAAAAAAGCAGGCTCATGGAAACC GTCATCCTGTC	This study
IHF β-up-R	GAACAAGGCAGAGATCACTCCTTGGTCATGGTTTTCCCTTC	This study
IHF β-down-F	GAGTGATCTCTGCCTTGTTC	This study
IHF β-down-R	GGGGACCACTTTGTACAAGAAAGCTGGGTACCATCAGAAA CGAAAGTAGTTC	This study
algB-seq-F	TGAGTTGTTCGTCGAGGATC	This study
algB-seq-R	GTCGATGCTGTCGAGTAC	This study
algB-up-F	GGGGACAAGTTTGTACAAAAAAGCAGGCTCAGATGATCAG AAAGGTGATAGC	This study
algB-up-R	CATGCTCATAGGCCGTACTGAGTGGTTTCCATCGTTGCTT	This study
algB-down-F	CAGTACGGCCTATGAGCATG	This study
algB-down-R	GGGGACCACTTTGTACAAGAAAGCTGGGTATCACGTCGAA ATCGCCTTC	This study
vfr-seq-F	GCATTATGCAGAGCCAATC	This study
vfr-seq-R	CAGTGATCGCCGAGATCAAG	This study
amrZ-SalI-F	TATATGTCGACAAATGCGCCCACTGAAACAGG	This study
amrZ-NotI-R	TATATGCGGCCGCAAGCTTTCAGGCCTGGGCCAGCT	This study
algR-SalI-F	TATATGTCGACAAATGAATGTCCTGATTGTCGA	This study
algR-NotI-R	TATATGCGGCCGCAAGCTTTCAGAGCTGATGCATCAGAC	This study
GST-algB-F	ACACAGGATCCATGGAAACCACTTCCGAAAAAC	This study
GST-algB-R	ACACAGAATTCATAGGCCGTACTGCTTGC	This study
P-algD_F	AAAAAGGTACCCCCGCAAGACTGATCTCCC	This study
P-algD_R	GAAAAGTTCTTCTCCTTTACGCATCGCATTCACCTCGATTG TTTG	This study
gfp_F (P-algD)	ATGCGTAAAGGAGAAGAACTTTTC	This study
gfp_R (P-algD)	GAGTCCAAGCTCAGCTAATTAAGC	This study
Tn7-glmS3	GAGAAGGTTTCCGAGCTGTTCGC	This study
Tn7R109	CAGCATAACTGGACTGATTTCAG	(7)
pGEX-seqF	GGGCTGGCAAGCCACGTTTGGTG	GE Healthsciences
pGEX-seqR	CCGGGAGCTGCATGTGTCAGAGG	GE Healthsciences
pET28b-seqF	TCGTCAGACTGTCGATGAAG	This study
pET28b-seqR	CTCAAGACCCGTTTAGAGGC	This study

Tn7L-in	ACTGGGTGTAGCGTCGTAAG	This study
Gfp-seq-int(+)	CCCGAAGGTTATGTACAGGAAAGA	This study
Seq-F-algD	CATCAAGTTGGTATCAAGTG	(8)
Pser-up	CGAGTGGTTTAAGGCAACGGTCTTGA	(10)
Pser-down	AGTTCGGCCTGGTGGAACAACTCG	(10)
qPCR-algD-F	TGTCGCGCTACTACATGCGTC	(11)
qPCR-algD-R	GTGTCGTGGCTGGTGATGAGA	(11)
qPCR-rpoD-F	ACAAGATCCGCAAGGTACTGAAG	(12)
qPCR-rpoD-R	CGCCCAGGTGCGAATC	(12)
qPCR-oprL-F	ATGGAAATGCTGAAATTCGGC	(1)
qPCR-oprL-R	ACCTTCACCGGAAGCATCG	(1)

References

1. Andersen JB, Hultqvist LD, Jansen CU, Jakobsen TH, Nilsson M, Rybtke M, Uhd J, Fritz BG, Seifert R, Berthelsen J, Nielsen TE, Qvortrup K, Givskov M, Tolker-Nielsen T. 2021. Identification of small molecules that interfere with c-di-GMP signaling and induce dispersal of *Pseudomonas aeruginosa* biofilms. npj Biofilms Microbiomes 7:59.
2. Stover CK, Pham XQ, Erwin AL, Mizoguchi SD, Warrener P, Hickey MJ, Brinkman FS, Hufnagle WO, Kowalik DJ, Lagrou M, Garber RL, Goltry L, Tolentino E, Westbrock-Wadman S, Yuan Y, Brody LL, Coulter SN, Folger KR, Kas A, Larbig K, Lim R, Smith K, Spencer D, Wong GK, Wu Z, Paulsen IT, Reizer J, Saier MH, Hancock RE, Lory S, Olson MV. 2000. Complete genome sequence of *Pseudomonas aeruginosa* PAO1, an opportunistic pathogen. Nature 406:959–964.
3. Rybtke MT, Borlee BR, Murakami K, Irie Y, Hentzer M, Nielsen TE, Givskov M, Parsek MR, Tolker-Nielsen T. 2012. Fluorescence-based reporter for gauging cyclic di-GMP levels in *Pseudomonas aeruginosa*. Appl Environ Microbiol 78:5060–5069.
4. Kessler B, de Lorenzo V, Timmis KN. 1992. A general system to integrate lacZ fusions into the chromosomes of gram-negative eubacteria: regulation of the Pm promoter of the TOL plasmid studied with all controlling elements in monocopy. Mol Gen Genet 233:293–301.

5. Hoang TT, Karkhoff-Schweizer RR, Kutchma AJ, Schweizer HP. 1998. A broad-host-range Flp-FRT recombination system for site-specific excision of chromosomally-located DNA sequences: application for isolation of unmarked *Pseudomonas aeruginosa* mutants. Gene 212:77–86.
6. Bao Y, Lies DP, Fu H, Roberts GP. 1991. An improved Tn7-based system for the single-copy insertion of cloned genes into chromosomes of gram-negative bacteria. Gene 109:167–168.
7. Lambertsen L, Sternberg C, Molin S. 2004. Mini-Tn7 transposons for site-specific tagging of bacteria with fluorescent proteins. Environ Microbiol 6:726–732.
8. Goltermann L, Tolker-Nielsen T. 2017. Importance of the Exopolysaccharide Matrix in Antimicrobial Tolerance of *Pseudomonas aeruginosa* Aggregates. Antimicrob Agents Chemother 61.
9. Almblad H, Harrison JJ, Rybtke M, Groizeleau J, Givskov M, Parsek MR, Tolker-Nielsen T. 2015. The cyclic AMP-Vfr signaling pathway in *Pseudomonas aeruginosa* is inhibited by cyclic di-GMP. J Bacteriol. 197:2190-200.
10. Hoang TT, Kutchma AJ, Becher A, Schweizer HP. 2000. Integration-proficient plasmids for *Pseudomonas aeruginosa*: Site- specific integration and use for engineering of reporter and expression strains. Plasmid 43:59–72.
11. Bragonzi A, Worlitzsch D, Pier GB, Timpert P, Ulrich M, Hentzer M, Andersen JB, Givskov M, Conese M, Doring G. 2005. Nonmucoid *Pseudomonas aeruginosa* expresses alginate in the lungs of patients with cystic fibrosis and in a mouse model. J Infect Dis. 192:410-419.
12. Jakobsen TH, van Gennip M, Phipps RK, Shanmugham MS, Christensen LD, Alhede M, Skindersoe ME, Rasmussen TB, Friedrich K, Uthe F, Jensen PØ, Moser C, Nielsen KF, Eberl L, Larsen TO, Tanner D, Høiby N, Bjarnsholt T, Givskov M. 2012. Ajoene, a sulfur-rich molecule from garlic, inhibits genes controlled by quorum sensing. Antimicrob Agents Chemother. 56:2314-2325.
